# Vitamin D Status and Its Influence on the Health of Preschool Children in Hangzhou

**DOI:** 10.3389/fpubh.2021.675403

**Published:** 2021-05-17

**Authors:** Zhaojun Chen, Xi Lv, Wensheng Hu, Xia Qian, Ting Wu, Yunxia Zhu

**Affiliations:** ^1^Department of Child Health Care, Hangzhou Women's Hospital (Hangzhou Maternity and Child Care Hospital), Hangzhou, China; ^2^Department of Teaching Office, Hangzhou First People's Hospital, Hangzhou, China

**Keywords:** vitamin D, preschool children, obesity, early children caries, respiratory tract infections

## Abstract

**Objective:** Vitamin D deficiency and insufficiency in children are global public health problems. However, few studies have focused on vitamin D status in healthy preschool children, especially in Asia. This study aimed to investigate vitamin D status and host-related factors in healthy preschool children in Hangzhou to analyze the impact of low vitamin D levels (<30 ng/mL) on health outcomes (obesity, early childhood caries, and respiratory tract infections).

**Methods:** A total of 1,510 healthy children aged 24–72 months from 15 kindergartens in Hangzhou were included. Data on the children's gender, age, body mass index (BMI), caries, and blood samples available for vitamin D analysis were collected from June to August 2018. A total of 325 children aged 36–48 months took part in a survey on the frequency of respiratory tract infections in the last year.

**Results:** The children's mean 25(OH)D level was 28.01 ± 7.29 ng/mL. A total of 11.4% of the children had vitamin D deficiency, and 52.6% had vitamin D insufficiency. Only 36.0% had vitamin D sufficiency. No significant difference was found by gender or BMI group. However, children in the obesity group had the highest prevalence of vitamin D deficiency and the lowest 25(OH)D levels. A significant negative correlation was found between the 25(OH)D level and child age (*r* = −0.144, *p* < 0.001). Regression analysis showed that the children’s 25(OH)D levels decreased by 0.17 ng/mL per month with age. In addition, children with low vitamin D levels might increase the risk of obesity and early childhood caries. Multiple linear regression indicated that the number of caries in children increased by 0.08 per 1-ng/mL decrease in the 25(OH)D level (*β* = −0.08, *p* < 0.001).

**Conclusion:** Vitamin D deficiency/insufficiency is a serious problem among healthy preschool children in Hangzhou. Public health policies or interventions should be implemented to ensure that preschool children have adequate vitamin D to reduce the risk of related diseases.

## Introduction

Vitamin D is a vital steroid hormone that is necessary for calcium and phosphorus absorption to maintain skeletal health (1). Vitamin D deficiency during childhood is known to cause growth retardation and nutritional rickets (2). Recently, an increasing number of studies have indicated that low levels of vitamin D may play an important role in the occurrence and development of extraskeletal diseases because of its immunoregulation and anti-inflammatory effects (3, 4), such as allergic diseases (5), respiratory illnesses (6), and obesity in children (7). Although there is no consensus on the association between vitamin D and related health outcomes, most specialists believe that sufficient vitamin D is essential and protective for children’s health.

Vitamin D is mainly synthesized in the skin. Generally, with unconstrained UVB exposure, the skin can generate adequate levels of vitamin D for human needs (8). However, an increasing number of systematic reviews have revealed that vitamin D deficiency/insufficiency in children is a global public health problem. In both high- and low-latitude regions, children show different degrees of vitamin D deficiency. A study in a high-latitude country (Irish, 51°N) found that more than 70% of children aged 2 years had vitamin D insufficiency (9). Another study from Uganda (2°N) also showed that 38.5% of children aged 5 years had low vitamin D levels (<30 ng/mL) and that 2.7% had vitamin D deficiency (10). Researchers have claimed that in addition to UVB exposure, vitamin D is also affected by genetic inheritance, religion, lifestyle, age, environmental pollution, and other risk factors. This may mean that vitamin D levels are more regional and population specific (11). Unfortunately, most studies on vitamin D levels in children have been conducted in high-latitude Western countries or populations with certain diseases, and few studies have focused on the vitamin D statuses of healthy children in Asia, although the number of related studies has gradually increased in recent years.

Hangzhou, the capital of Zhejiang Province, is located in southeastern China at a latitude of 30°33′ N. In recent years, this rapidly growing city has experienced severe air quality issues (12). Under these conditions, the synthesis of vitamin D is likely to be greatly diminished. However, no studies have investigated the vitamin D statuses of healthy children in Hangzhou in the last 5 years. Unlike infants, who were recommended to receive supplements of 400 international units of vitamin D per day from 2 weeks to 2 years by the Chinese Medical Association, and school-age children, who have plenty of outdoor activity time according to school regulations, preschool children often seem to be neglected. Therefore, in this study, our aims were to investigate the vitamin D statuses of healthy preschool children in Hangzhou and evaluate the host-related influential factors of vitamin D deficiency and insufficiency. Additionally, considering that obesity, early childhood caries (ECC), and respiratory tract infections are diseases with high incidence rates in preschool children, we also analyzed the influence of vitamin D deficiency/insufficiency on these health outcomes.

## Materials and Methods

### Survey and Participants

A total of 1,510 children aged 24–72 months from 15 kindergartens in Hangzhou were scheduled to attend the child health care department of Hangzhou Women’s Hospital for prekindergarten health examinations from June to August 2018. Data including basic information (gender, age), growth development (height, weight, caries), and serum 25(OH)D levels were collected. Children were assessed on the status of caries by children’s health care doctors. Children with no decayed tooth were classified into caries-free group (caries “No” group), and others were classified into caries group (caries “Yes” group). Body mass index (BMI) was calculated as body mass in kilograms divided by height in meters squared (kg/m^2^). Obesity was defined as BMI in the ≥95th percentile, overweight was defined as BMI between the 85th and 94th percentiles, normal weight was defined as BMI between the 5th and 84th percentiles, and underweight was defined as BMI in less than the fifth percentile. Blood samples were collected on the day of physical examination, and serum 25(OH)D level was detected within 24 h.

Thirty percent of the participants aged 36–48 months took part in a survey. A total of 345 questionnaires were sent out, and 325 (94.2%) were collected. The questionnaire had four items, including on the frequency of upper respiratory tract infection (URTI) in the last year, the frequency of pneumonia in the last year, the frequency of trachea bronchitis in the last year, and whether the child had taken vitamin D in the last 30 days. All the questionnaires were filled in by the guardian of the child. It is suggested that the guardian should refer to the frequency of illness recorded in the child’s medical record last year. Recurrent respiratory infection was defined as the frequency of URTI ≥6 times/year or the frequency of trachea bronchitis ≥2 times/year or the frequency of pneumonia ≥2 times/year in the past year.

In this cross-sectional study, children with any illness that might affect hydroxylation of vitamin D, calcium, and phosphorus metabolism (rickets or hypocalcemia or abnormal liver or renal function) were excluded. Ethical approval (No. 20180413) was granted by Hangzhou Women’s Hospital Ethics Committee, and informed consent was obtained from the parents of all participants.

### Serum 25(OH)D Level

Fasting blood samples were centrifuged at 1,000 revolutions/min for 10 min after collection, and then the serum was stored at −20°C until analysis. Before analysis, 100 μL of serum was mixed with 200 μL of methanol with internal standard and then vibrated for 5 min. The internal standards of vitamin D_2_ and D_3_ were 25-hydroxyvitamin D2–d3 and 25-hydroxyvitamin D3–d6, respectively. The supernatants were collected and extracted with 600 μL of n-hexane and then analyzed by liquid chromatography–mass spectrometry. The vitamin D level was the sum of serum 25(OH)D_2_ and serum 25(OH)D_3._

Vitamin D status was determined based on the serum 25(OH)D level and categorized into three groups: vitamin D deficiency [serum 25(OH)D ≤ 20 ng/mL], vitamin D insufficiency (20–30 ng/mL), and vitamin D sufficiency ≥30 ng/mL).

### Statistical Analyses

We used SPSS version 21.0 for all analyses. Categorical variables were summarized as the number and percentage, and continuous variables were summarized as the mean and standard deviation (SD). Independent-samples *t*-tests (gender, caries, and recurrent respiratory infection) and one-way analysis of variance (age, BMI) were used to compare the differences in vitamin D levels among groups. Pearson χ^2^ test was used to compare the differences in vitamin D status among groups. Spearman correlation analysis was used to examine the correlations between vitamin D level and child age and between vitamin D level and number of caries. Multiple linear regression was used to predict the effect of vitamin D level on caries, adjusted for gender, age, and BMI. Two-tailed *p* < 0.05 was considered statistically significant.

## Results

### Participant Characteristics

A total of 1,510 healthy children from 15 kindergartens in Hangzhou City participated in this cross-sectional study. A total of 54.2% of the children were boys, and the remaining children were girls. Their mean age was 44 ± 8.2 months (range = 24–72 months), and they were divided into four groups according to their ages: Group_24−36*M*_ (24–36 months, *n* = 142), Group_36−48*M*_ (36–48 months, *n* = 1,150), Group_48−60*M*_ (48–60 months, *n* = 128), and Group_60−72*M*_ (60–72 months, *n* = 90). Most of the children (87.2%) had a BMI within the normal range, but 1.4, 8.9, and 2.5% of them were underweight, overweight, and obese, respectively. In addition, 24.37% of the children (*n* = 368) had ECC. The characteristics of the participants are shown in Table 1.

**Table 1:** The influence of host-related factors (gender, age, BMI) on serum 25(OH)D levels and vitamin D status in children.

**Host-related factor (*****n*****, %)**		**25(OH)D level, mean ± SD (ng/mL)**	***p***	**VD status**, ***n*** **(%)**			***p***
				**Deficiency (<20 ng/mL)**	**Insufficiency (20–30 ng/mL)**	**Sufficiency (≥30 ng/mL)**	
Gender	Boy (*n* = 818, 54.17%)	27.97 ± 7.39	0.791	96 (11.74%)	435 (53.18%)	287 (35.09%)	0.687
	Girl (*n* = 692, 45.83%)	28.07 ± 7.18		76 (10.98%)	359 (51.87%)	257 (37.14%)	
Age	24–36 months (*n* = 144, 9.54%)	29.44 ± 7.30	**<0.001**	6 (4.17%)	79 (54.86%)	57 (39.58%)	**<0.001**
	36–48 months (*n* = 1,149, 76.09%)	28.49 ± 7.13		110 (9.57%)	607 (52.83%)	433 (37.68%)	
	48–60 months (*n* = 127, 8.41%)	25.38 ± 6.68		26 (20.47%)	66 (51.96%)	36 (28.34%)	
	60–72 months (*n* = 90, 5.96%)	23.30 ± 7.29		30 (33.33%)	42 (46.67%)	18 (20.00%)	
BMI	Underweight (*n* = 21, 1.39%)	27.17 ± 7.41	0.159	4 (19.04%)	9 (42.85%)	8 (38.09%)	0.077
	Normal (*n* = 1,317, 87.21%)	28.16 ± 7.28		138 (10.47%)	703 (53.37%)	476 (36.14%)	
	Overweight (*n* = 135, 8.94%)	27.41 ± 7.33		21 (15.55%)	65 (48.15%)	49 (36.30%)	
	Obesity (*n* = 37, 2.45%)	25.51 ± 7.34		9 (24.32%)	17 (45.95%)	11 (29.73%)	
Total	1,510	28.01 ± 7.29		172 (11.39%)	794 (52.58%)	544 (36.03%)	

*BMI, body mass index; SD, standard deviation. Bold means statistical significance difference (P < 0.05)*.

### Host-Related Factors Influencing Vitamin D Level/Status

The serum 25(OH)D concentrations of all 1,510 children were measured. The mean 25(OH)D levels of the children were 28.01 ± 7.29 ng/mL, and the levels ranged from 8.5 to 62.4 ng/mL. A total of 52.6% of children had vitamin D insufficiency, and another 11.4% had deficiency. Only 36.0% of them showed vitamin D sufficiency.

We found no significant difference in the mean vitamin D level or vitamin D status between boys and girls (*p* > 0.05). Similarly, there were also no significant differences among the different BMI groups (*p* > 0.05). However, it is worth noting that the 25(OH)D levels of children in the normal group were highest (28.16 ± 7.3 ng/mL), and those of the obese group were lowest (25.51 ± 7.4 ng/mL). The prevalence of vitamin D deficiency was also the highest in the obesity group (24.3%) and the lowest in the normal group (10.5%).

**Figure 1:**
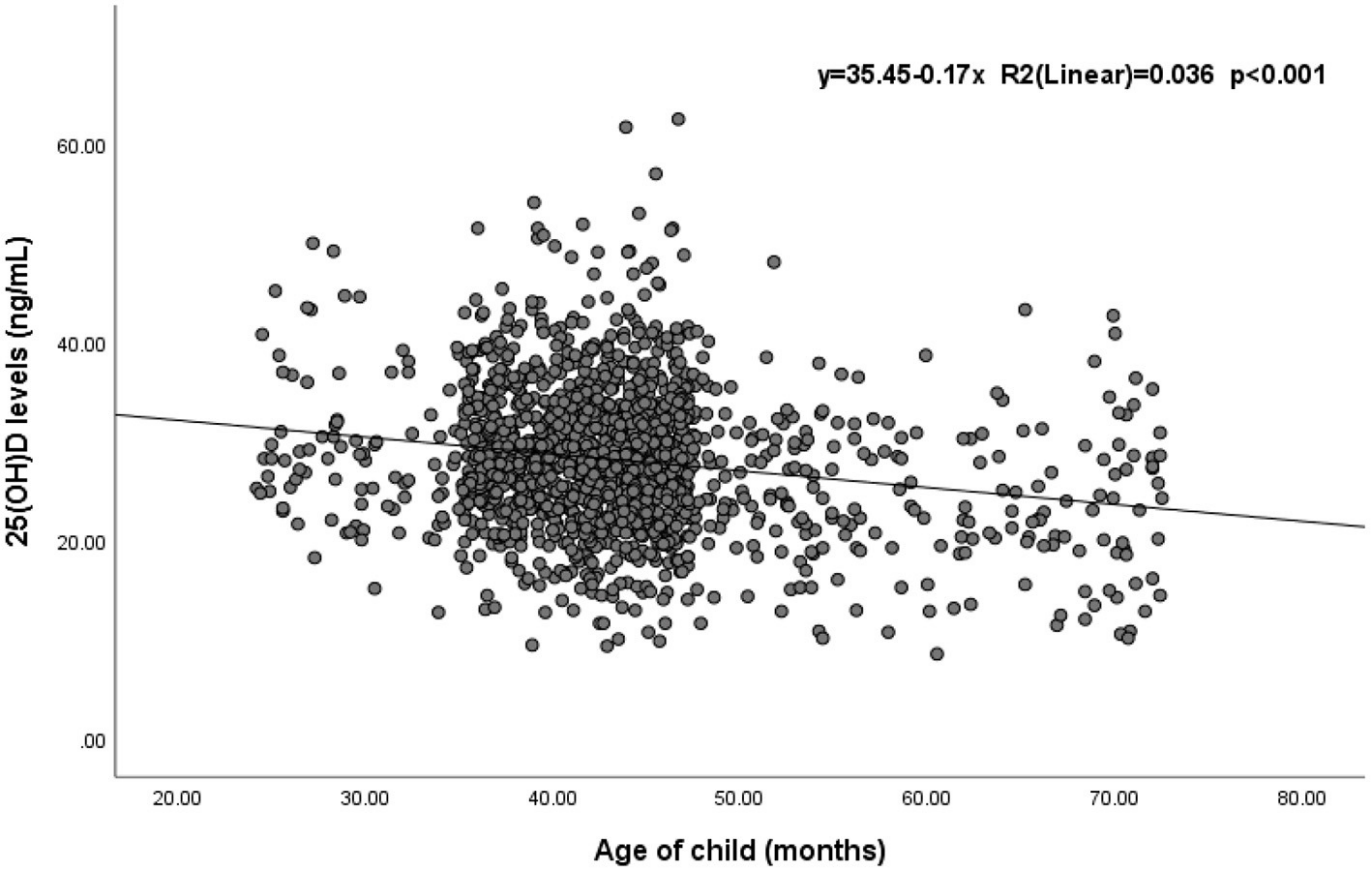
The effect of age on 25(OH)D levels in children (*n* = 1,510).

Significant differences in the 25(OH)D level and vitamin D status were found among different age groups (*p* < 0.05). Children aged 25–36 months had the highest 25(OH)D level (29.44 ± 7.3 ng/mL), and the mean level decreased with age. Similarly, the prevalence of vitamin D sufficiency decreased from 40.1 to 20.0% with age (Table 1). Spearman correlation analysis suggested a statistically significant negative correlation between 25(OH)D levels and children’s age (*r* = −0.144, *p* < 0.001) (Supplementary Table 1). Additionally, a simple linear regression was calculated to determine the effect of age on 25(OH)D levels in children (Figure 1). The coefficient of determination (*R*^2^) was 0.036, meaning that 3.6% of the variance in 25(OH)D levels was explained by children’s age. The regression equation showed that the 25(OH)D level was equal to −0.17 (age) + 35.45 (ng/mL) (*p* < 0.001; *R*^2^ = 0.036) and indicated that children’s 25(OH)D levels decreased by 0.17 ng/mL at every month of age.

### Correlation of Vitamin D Level/Status and Related Health Outcomes

#### Obesity

Compared with those of non-obese children, the 25(OH)D levels of obese children were significantly lower (*p* = 0.035) at 28.07 ± 7.28 and 25.51 ± 7.34 ng/mL, respectively. Moreover, the prevalence of obesity in the group with vitamin D deficiency was significantly higher than that in the groups with vitamin D insufficiency and sufficiency (*p* = 0.043), at 5.23, 2.14, and 2.02%, respectively (Table 2).

**Table 2:** Correlation of serum 25(OH)D level/vitamin D status and related health outcomes.

**Health outcome**		**Group**	**No. (%)**	**25(OH)D level, mean ± SD (ng/mL)**	***p***	**VD status**, ***n*** **(%)**			***p***
						**Deficiency (<20 ng/mL)**	**Insufficiency (20–30 ng/mL)**	**Sufficiency (≥30 ng/mL)**	
Obesity		No	1,473 (97.55)	28.07 ± 7.28	**0.035**	163 (94.77)	777 (97.86)	533 (97.98)	**0.043**
		Yes	37 (2.45)	26.98 ± 7.43		9 (5.23)	17 (2.14)	11 (2.02)	
Early childhood caries		No	1,142 (75.63)	28.35 ± 7.22	**0.002**	121 (70.35)	588 (74.05)	433 (79.60)	**0.016**
		Yes	368 (24.37)	26.98 ± 7.43		51 (29.65)	206 (25.95)	111 (20.40)	
Respiratory tract infections	Upper respiratory tract infection	No	9 (2.77)	31.98 ± 9.89	0.216	1 (2.94)	3 (1.86)	5 (3.84)	0.590
		Yes	316 (97.23)	28.89 ± 7.31		33 (97.06)	158 (98.14)	125 (96.16)	
	Trachea bronchitis	No	260 (80.00)	28.98 ± 7.46	0.975	26 (76.47)	130 (80.75)	104 (80.00)	0.852
		Yes	65 (20.00)	28.95 ± 7.14		8 (23.53)	31 (19.25)	26 (20.00)	
	Pneumonia	No	300 (92.31)	28.91 ± 7.32	0.580	31 (91.18)	149 (92.55)	120 (92.31)	0.964
		Yes	25 (7.69)	29.76 ± 8.23		3 (8.82)	12 (7.45)	10 (7.69)	
	Recurrent respiratory infection	No	286 (88.00)	28.67 ± 7.41	0.048	32 (94.11)	143 (88.82)	111 (85.38)	0.341
		Yes	39 (12.00)	31.65 ± 6.92		2 (5.89)	18 (11.18)	19 (14.62)	

*No., number of children; SD, standard deviation. Bold means statistical significance difference (P < 0.05)*.

#### Early Childhood Caries

The prevalence of ECC among the 1,510 children was 24.4%, and the child with the most severe ECC had 14 decayed teeth. There were significant differences in the vitamin D levels and vitamin D statuses of children with and without ECC (*p* = 0.002). Compared with children who had vitamin D sufficiency (20.40%), children with vitamin D insufficiency and deficiency had a significantly higher prevalence of ECC (*p* = 0.016), at 25.95 and 29.65%, respectively (Table 2).

**Figure 2:**
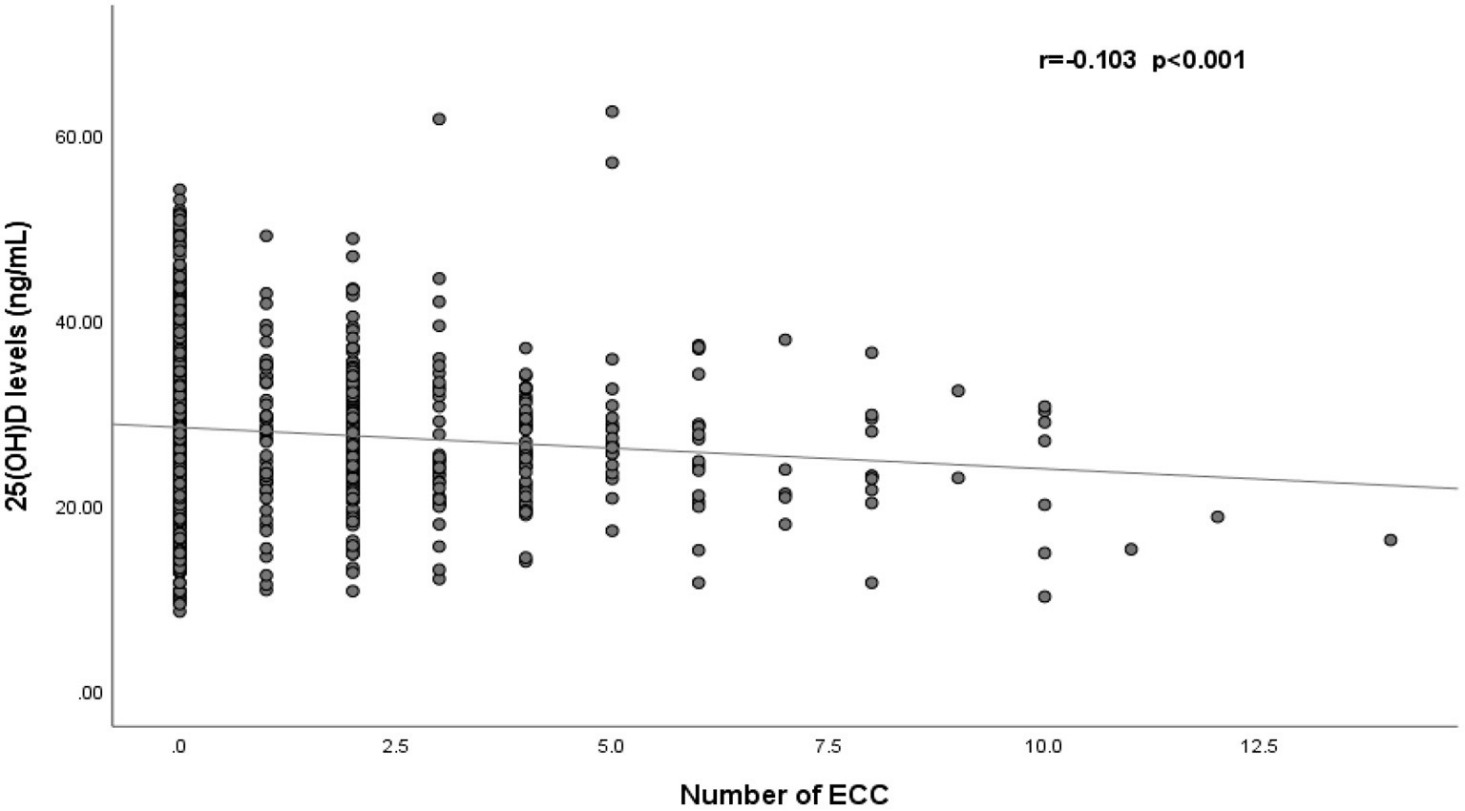
Correlation of serum 25(OH)D levels and the number of early childhood caries (*n* = 1,510). ECC, early childhood caries.

Figure 2 shows a negative correlation between 25(OH)D levels and the number of caries in children (*r* = −0.103, *p* < 0.001). After adjustment for gender, age and BMI, the results of multiple linear regression indicated that the number of caries in children increased by 0.08 per 1-ng/mL decrease in 25(OH)D (β = −0.08, *p* < 0.001) (Supplementary Table 2).

#### Respiratory Tract Infections

The results of the questionnaire survey show that among the 325 children surveyed, 316, 65, 25, and 39 children had URTI, trachea bronchitis, pneumonia, and recurrent respiratory infections in the last year, respectively.

For children who had URTI, trachea bronchitis, or pneumonia in the last year, their vitamin D levels were similar to those of healthy children. Interestingly, however, compared with healthy children, children who had recurrent respiratory infections in the last year had relatively higher vitamin D levels (*p* = 0.048), at 28.67 ± 7.41 vs. 31.65 ± 6.92 ng/mL, respectively. But after adjustment for gender, age, and BMI, the results of multiple linear regression indicated there were also no significant differences of vitamin D levels between children with recurrent respiratory infections and healthy children (*p* = 0.059) (Supplementary Table 3). In addition, no significant differences were found regarding the probability of children with different vitamin D statuses (sufficiency, insufficiency, and deficiency) suffering from respiratory tract infections (Table 2).

## Discussion

Vitamin D deficiency and insufficiency are global public health problems. Although the importance of vitamin D to health has been a concern in recent years, there are few studies on vitamin D in preschool children. Table 3 summarizes the articles published in the last 5 years on vitamin D levels and risk factors for preschool children in PubMed, which indicate that vitamin D deficiency/insufficiency is a serious problem in preschool children in most parts of the world.

**Table 3:** Prevalence and risk factors for low vitamin D (vitamin D deficiency and insufficiency) in preschool children worldwide.

**Country (references)**	**No. of population**	**Investigated time**	**Age**	**Mean 25(OH)D level (ng/mL)**	**Low vitamin D**		**Vitamin D sufficiency (≥30 ng/mL)**	**Risk factors**			
					**Deficiency (<20 ng/mL)**	**Insufficiency (20–30 ng/mL)**		**Gender**	**Age**	**BMI**	**Other factors**
Oman (33)	2,531	Dec 2016 to Apr 2017	6–59 months	7.44 (7.20–7.64)	53.80%	46.20%		G > B	NS	—	—
Bahrain (14)	257	Sep to Oct 2016	1 month to 5 years	—	67.30%	23.00%	9.70%	G > B	Negative correlation	—	—
Japan (34)	574	May 2010 to Nov 2013	36 months	23.5 ± 6.1	29.60%	70.40%		NS	—	—	Season; daily UVB radiation, daily outdoor play time
China (1)	804	Jan to Dec 2016	3–6 years	20.34	48.10%	42.20%	9.70%	NS	Negative correlation	—	Season
China (11)	2,280	Mar 2014 to Feb 2015	2–6 years	31.0 ± 10.28	15.10%	34.50%	50.40%	NS	Negative correlation	—	Socioeconomic environment
China (13)	2,872	Oct to Dec 2015	3–5 years	—	19.50%	39.60%	40.90%	—	—	Overweight and obesity	—
China^*^	1,510	Jun to Aug 2018	24–72 months	28.01 ± 7.29	11.4%	52.6%	36.0%	NS	Negative correlation	Obesity	—
Norway (2)	212	Jan to Feb 2015	4–6 years	24.28 ± 5.52	18.90%	65.50%	15.60%	NS	—	Overweight	—
Southern Croatia (35)	260	Mar to Apr 2017	5–6 years	18.62 ± 8.06	58.00%	29.00%	13.00%	G > B	—	—	—
Kamba tribe in Kenya (36)	259	Mar 2013	3–5 years	33(32.28, 33.72)	0.80%	99.20%		—	—	—	—
Maasai tribe in Kenya (36)	174	Mar 2013	3–5 years	38.28(37.24, 39.32)	0.00%	100%		—	—	—	—
Northern Iran (20)	390	Jun 2015 to Apr 2016	30–72 months	—	30.50%	38.50%	31.00%	NS	NS	Overweight	Season
Canada (30)	279	Between the late summer and fall of 2007 and 2008	3–5 years	19.4	12.50%	66.67% (10–30 ng—mL)	20.80%	—	—	—	—
New Zealand (37)	1,329	Late winter to spring 2012	2–5 years	20.8 ± 7.6	48.00%	41.00%	11.00%	G > B	—	—	Non-European ethnicities; olive-dark skin color; not taking VD supplements; mother’s educational level; living in more deprived households

*No., number; BMI, body mass index; G > B, girls have a significantly higher risk for low vitamin D; NS, no significant difference; —, No available data*.

* *Present study*.

In this study, we found that more than half of the preschool children (52.6%) in Hangzhou had vitamin D insufficiency. Compared with previous reports on preschool children in Asia, our study showed that preschoolers in Hangzhou had the lowest rate of vitamin D deficiency (11.4%), but the vitamin D sufficiency rate was lower than that in other cities in East China (Changsha and Wenzhou City) (11, 13). This difference may be related to the economy, latitude, and eating habits. Hangzhou is affluent, and the one-child policy in China makes parents eager to offer nutritious food to their babies, which may lead to a low rate of vitamin D deficiency among preschool children in Hangzhou. However, compared with the latitudes of Wenzhou and Changsha (27°03′ N and 27°51′ N, respectively), the latitude of Hangzhou is 2 degrees higher, at 30°33′ N. The higher latitude means more sun exposure and vitamin D synthesis. In addition, the diet of Wenzhou is dominated by ocean fish all year, which can provide children with sufficient vitamin D. It should be noted that our study may have overestimated the children’s overall vitamin D levels because it was conducted in the summer (1). Thus, more attention should be paid to the vitamin D statuses of preschool children, and supplementation with appropriate vitamin D is necessary in Hangzhou.

Consistent with many studies (1, 2), we found no significant difference in the vitamin D status between boys and girls. However, a study in Bahrain showed that the mean 25(OH)D levels of girls were significantly lower than those of boys and that girls had a higher prevalence of vitamin D deficiency (14). Similarly, a study in Saudi Arabia also reported that vitamin D deficiency was more serious among females than males (15). The difference might be due to different cultural factors and religious behaviors. Girls in Muslim countries with traditional dress have less sun exposure than boys, and boys tend to play outside and produce more vitamin D (14, 16).

Young age of children has often been found to be a potential risk factor for vitamin D deficiency and insufficiency. Most studies have shown significant differences in vitamin D levels among children of different ages, with the prevalence of vitamin D deficiency increasing with age (1, 11). These studies paid more attention to school-age children because of their less time spent outdoors (17) or because the age span of research participants was relatively large (e.g., 0 months to 16 years) (18). Considering that few studies have been conducted on preschoolers, in this study, our subjects were children aged 25–72 months who were divided into 12-month age groups. Previous research showed that there may be age-related functional alterations of gastrointestinal change based on the efficiency of vitamin D absorption (19), and we found the same results: the mean 25(OH)D levels and the prevalence of vitamin D sufficiency decreased with age. In addition, Spearman correlation analysis also suggested a significant inverse association between 25(OH)D levels and age. Even so, however, the association and mechanism between vitamin D and age are still unconfirmed (14). Further studies should be performed to determine how vitamin D status is affected by children’s age.

An inverse association between BMI and 25(OH)D levels among children has been widely reported (2, 20). A study conducted with preschoolers aged 2.5–6 years showed that after adjustment for gender, age, place of residence, season, and sun exposure, BMI continued to be associated with an increased odds ratio of vitamin D deficiency, and it was an independent predictor of vitamin D deficiency (20). Another study in Norway also indicated that preschoolers aged 4–6 years who were overweight had significantly lower 25(OH)D levels than non-overweight preschoolers, showing that overweight status increased the risk for vitamin D deficiency (2). In our study, however, no significant difference in the 25(OH)D level or vitamin D status was found among the four BMI groups. It is worth noting that the 25(OH)D levels of the children in the normal group were highest (28.16 ± 7.3 ng/mL), and those of the children in the obesity group were lowest (25.51 ± 7.4 ng/mL). Meanwhile, the prevalence of vitamin D deficiency was also highest in the obesity group (24.3%) and lowest in the normal group (10.5%). This finding may be due to the lack of power for this hypothesis (only 2.5% of the preschoolers in this study were obese) (2). Thus, we then divided the children into obesity group and non-obesity group, and the results showed that the mean 25(OH)D levels of the two groups were significantly different. Therefore, more attention should be paid to obese children for vitamin D supplementation.

Does obesity decrease vitamin D levels, does vitamin D deficiency promote obesity, or do they interact? On the one hand, researchers believe vitamin D bioavailability in obese individuals is decreased because of vitamin D deposition in body adipose tissue and altered expression levels of vitamin D–metabolizing enzymes (2, 7, 17). Moreover, obese children are less likely to engage in outdoor activities, which results in reduced vitamin D synthesis (21). On the other hand, studies found that vitamin D regulated multiple aspects of adipose biology, such as adipogenesis and metabolic and endocrine function of adipose tissues (7). This may be the reason for obesity and metabolic diseases caused by vitamin D deficiency. To date, however, the association between vitamin D deficiency and adiposity is still unclear (22). In our study, we analyzed the effect of vitamin D status on obesity. We found that compared with vitamin D sufficiency, vitamin D insufficiency had a slight effect on obesity in preschool children; only when vitamin D was <20 ng/mL did the prevalence of obesity increase significantly. Therefore, 20 ng/mL may be the vitamin D cutoff value for preventing obesity.

In addition to obesity, we also analyzed the possible association between vitamin D and ECC. We found a significant negative correlation between the 25(OH)D level and the number of caries. After adjustment for gender, age, and BMI, multiple linear regression indicated that the number of caries increased by 0.08 per 1-ng/mL decrease in 25(OH)D. Although few studies have focused on the relationship between vitamin D and ECC, especially in Asia (16), our results are still supported by some studies (23, 24). A cross-sectional study in the United States indicated a significant association between vitamin D and ECC in children aged 1–6 years and showed that children with suboptimal vitamin D (serum 25(OH)D level <75 nmol/L) were twice as likely to have ECC than children with optimal levels (23). Chhonkar et al. also showed that vitamin D deficiency was a risk factor both for the incidence and severity of ECC in children aged 3–6 years (24). This relationship between vitamin D and ECC may be explained by the mineralization and antibacterial function of vitamin D. On the one hand, vitamin D deficiency can induce defective tooth mineralization, resulting in dentin and enamel defects, which can increase the risk of dental caries (16). On the other hand, vitamin D can induce certain antimicrobial peptides that protect against oral pathogens, such as defensins and cathelicidins (25). Remarkably, recent studies have suggested that maternal 25(OH)D deficiency may increase the risk of enamel hypoplasia and the rates of ECC in infants (26). Although the mechanism of the effect of vitamin D on ECC is unclear, researchers tend to believe that sufficient vitamin D early in life (including pregnancy) is a promising preventive agent against ECC (27).

Considering the immunomodulatory properties of vitamin D, several studies have attempted to determine the association between vitamin D and infectious diseases of the respiratory system, such as acute otitis media, bronchiolitis, and pneumonia (28). A study from Maharashtra showed a significantly negative correlation between serum 25(OH)D concentrations and episodes of URTI in school-aged children and indicated that children with vitamin D sufficiency had fewer URTI episodes and shorter URTI durations than children with vitamin D insufficiency (29). Another study also indicated that low serum 25(OH)D levels could increase the risk of acute lower respiratory tract infections in newborn babies (6). In this study, we tried to understand the possible link between serum 25(OH)D levels and the occurrence of respiratory tract infection (URTI, trachea bronchitis, pneumonia, and recurrent respiratory infection) in preschoolers. However, our results did not suggest a significant association between lower 25(OH)D levels and the occurrence of all respiratory tract infections we investigated, which was in line with some studies (30). Sudfeld et al. reported no association between vitamin D status and the incidence of URTI and acute lower respiratory tract infection in children aged 6 months (31). A randomized, double-blind, placebo-controlled trial indicated that vitamin D–calcium supplementation exerted no effect on the occurrence of infections compared with the placebo (32). All of these conflicting results may have been induced by several confounding factors, such as seasonal and racial differences, populations of different ages, and different cutoffs of vitamin D (3). In this study, the number of respiratory infections in the last year was recalled by the children’s parents, and we cannot rule out the effect of recall bias on the results.

## Conclusions

Our study suggested a high prevalence of low vitamin D levels (vitamin D insufficiency and deficiency) in preschool children in Hangzhou. The age and BMI of children possibly affected their vitamin D levels. More attention should be paid to older children and obesity children for vitamin D supplement. In addition, vitamin D deficiency increased the risk of obesity and ECC in preschool children, which means keeping sufficient vitamin D is essential for children’s health.

## Data Availability Statement

The raw data supporting the conclusions of this article will be made available by the authors, without undue reservation.

## Ethics Statement

The studies involving human participants were reviewed and approved by Hangzhou Women’s Hospital Ethics Committee. Written informed consent to participate in this study was provided by the participants’ legal guardian/next of kin.

## Author Contributions

ZC, XQ, and TW were responsible for data collection. ZC was responsible for writing the first draft of the article and analyzing the data with XL. YZ and WH revised and finalized the article. All authors contributed to the article and approved the submitted version.

## Conflict of Interest

The authors declare that the research was conducted in the absence of any commercial or financial relationships that could be construed as a potential conflict of interest.
