# Supplementary material for: Vitamin D Status and Its Influence on the Health of Preschool Children in Hangzhou
Source: Front Public Health. 2021 May 17;9:675403. doi: 10.3389/fpubh.2021.675403 (PMC8165265; doi:10.3389/fpubh.2021.675403)
Supplement: Supplementary file 1 [file Data_Sheet_1.docx]

| Supplementary Table 1. Correlation coefficient between host-related factors and serum 25(OH)D levels | | |
| --- | --- | --- |
|  | 25(OH)D level | |
|  | Correlation coefficient | *P* value |
| Gender | 0.014 | 0.593 |
| Age | -0.144 | **<0.001** |
| BMI | -0.023 | 0.367 |
| Number of caries | -0.103 | **<0.001** |

BMI：Body Mass Index.

Supplementary Table 2. Multiple linear regression coefficient between the number of caries and related factors

| Model | Multiple linear regression coefficient | | *t* value | *P* value |
| --- | --- | --- | --- | --- |
|  | β | S.E. |  |  |
| Constant | 9.197 | 1.191 | 7.721 | <0.001 |
| 25(OH)D levels | -0.08 | 0.011 | -7.217 | <0.001 |
| Gender | -0.932 | 0.167 | -5.579 | <0.001 |
| BMI | -0.24 | 0.068 | -3.552 | <0.001 |
| Age | 0.032 | 0.009 | 3.54 | <0.001 |

S.E.: Standard Error; BMI：Body Mass Index.

Supplementary Table 3. Multiple linear regression coefficient between the occurrence of recurrent respiratory infections and related factors

| Model | Multiple linear regression coefficient | | *t* value | *P* value |
| --- | --- | --- | --- | --- |
|  | β | S.E. |  |  |
| Constant | 1.721 | 0.345 | 4.987 | <0.001 |
| 25(OH)D levels | -0.05 | 0.002 | -1.893 | 0.059 |
| Gender | 0.011 | 0.036 | 0.312 | 0.755 |
| BMI | 0.011 | 0.014 | 0.792 | 0.429 |
| Age | 0.003 | 0.005 | 0.492 | 0.623 |

S.E.: Standard Error; BMI：Body Mass Index.
